# 425. Sustained Control and Prevention of COVID-19 Outbreaks in Detroit Skilled Nursing Facilities

**DOI:** 10.1093/ofid/ofab466.625

**Published:** 2021-12-04

**Authors:** Seema Joshi, Abigail Manning, James Bivins, Samia Arshad, Amen Agbonze, Anita M Berger, Bonnie Czander, Gonzalo Gonzalez, Jessica H Heinonen, Keith Klama, Lakita L Lamb, Helina Misikir, Alexis Adams, Micah Anderson, Madelyn Morley, Paul E Kilgore, Marcus Zervos, Michael Mossing, Katerina Stylianou, Najibah K Rehman

**Affiliations:** 1 Henry Ford Hospital, Detroit, Michigan; 2 Detroit Health Department, Dexter, Michigan; 3 Henry Ford Health System, Detroit, Michigan; 4 CDCF, Northville, Michigan; 5 Detroit Health Department & CDC Foundation, Ann Arbor, Michigan; 6 Arrow Strategies, Detroit, Michigan; 7 CDC Foundation, Detroit, Michigan; 8 Eugene Applebaum College of Pharmacy and Health Sciences, Detroit, Michigan

## Abstract

**Background:**

Nursing home residents, a vulnerable population, experienced an extraordinary surge of COVID-19 cases and deaths at the beginning of the pandemic. Multidisciplinary collaboration from the Detroit Health Department (DHD), academic centers, along with interim guidance from the CDC provided a structured approach to control SARS-CoV-2 in Detroit skilled nursing facilities (SNF). We aim to describe this model.

**Methods:**

There were 26 SNF prioritized by the DHD over a 13-month period from 3/2020 - 4/2021. Testing for SARS-CoV-2 occurred biweekly, on average, at each facility for staff and residents. Any staff or resident cases were investigated by a specialized investigations team to determine outbreak status. Any resident that was identified as positive for SARS-CoV-2 was moved to a designated in-house quarantine unit or specific COVID-19 designated nursing homes within the City of Detroit, and cohorting guidance was provided. Facilities were evaluated for environmental controls, PPE provided as needed and infection prevention guidance was provided. COVID-19 vaccination was conducted by pharmaceutical chains or the DHD and vaccine education sessions were conducted for nursing home staff and residents.

**Results:**

On average, SNF facilities served a total of 2,262 residents (2031-2367 range) and employed a total of 2,965 staff (1034-3124 range) during the period from 7/2020 - 4/2021. SARS-CoV-2 cases overall for Michigan and Detroit are shown in Figure 1. In SNF facilities, cases ranged from zero to 279 cases in residents and zero to 115 cases per week in staff (Figure 1). Beginning 3/2020, the majority of cases were residents, whereas after 10/2020, staff cases exceeded resident cases. Immunization rates were 63% (partial) and 58% (complete) for residents, and 26% and 23% for staff, respectively. Measures to reduce vaccine hesitancy included organized education sessions, messaging from trusted leaders and organized mass vaccination schedules.

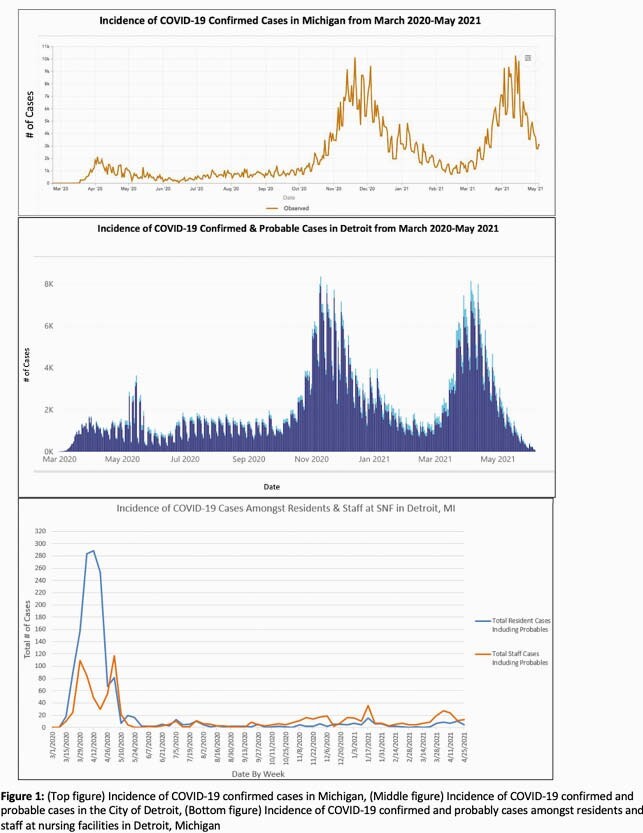

**Conclusion:**

We describe the effectiveness of multidisciplinary interventions to control dissemination, morbidity and mortality of SARS-CoV-2 amongst SNF residents in Detroit. We emphasize the continued need to address vaccine hesitancy and importance of this model as successful interventions to decrease infection rates.

**Disclosures:**

**Paul E. Kilgore, M.D., M.P.H.**, **Johnson and Johnson (Janssen**) (Grant/Research Support, Scientific Research Study Investigator)**Moderna** (Grant/Research Support, Scientific Research Study Investigator) **Marcus Zervos, MD**, **contrafect** (Advisor or Review Panel member)**janssen** (Grant/Research Support)**merck** (Grant/Research Support)**moderna** (Grant/Research Support)**pfizer** (Grant/Research Support)**serono** (Grant/Research Support)

